# Children—Their Requirements, Moral and Physical, Essential to a Healthful Development—Influences Which Have a Salutary or Baneful Effect, Etc.

**Published:** 1860-12

**Authors:** 


### ARTICLE II.

*Children—Their Requirements, Moral and Physical, Essential to a Healthful Development—Influences which have a Salutary or Baneful Effect etc.*

There is, perhaps, no subject more worthy the attention of the readers of the Hygiene department of your Journal, especially those who are parents, than the proper treatment of children. The limits which I have assigned myself in the present article, forbid that I consider the nature of those influences which may be exerted to act advantageously or otherwise upon the unborn child. It would be interesting to notice the custom prevalent with some of the most cultivated ancients, and which at present exists among some of the orientals, of surrounding a woman during the period of jestation with agreeable objects—delighting her ear with music, her eye with paintings—her imagination with pleasing stories, heroic adventures, etc., or to recount some of the many historical and traditional instances which establish the existence of a popular belief, in almost every age of man's history, as to the permanent character of mental impressions which are supposed to affect the child, through the mind of the mother, *in utero.* The mother of Napoleon the 1st is supposed to have impressed him with a martial spirit by accompanying her husband through scenes calculated to impress her mind with a war-like feeling and calling for the exercise of military heroism, while *enciente.* Without pausing to discuss these mooted questions, let us consider the requirements of the babe after it has reached the breast of its mother. Almost the very first requirement of the new-born babe is nourishment, which I maintain is appropriately furnished *alone* by the parent. I propose to give a few simple rules which any intelligent mother may understand, and which if strictly adhered to will insure health to the child nine cases out of ten, and much comfort to the parent. As a general thing the child should have its own mother's milk, and that *exclusively* until after the appearance of teeth; when the infant is in a thriving condition a little panada made with a soda craker—a little well boiled rice (the rice should be cooked to a pulp), a little finely minced breast of chicken, quail or turkey may also be allowed it, gradually increasing the variety and quantity of food as it grows older, and as the food is found to agree with it. Very little feeding is necessary or prudent until it is twelve months old, when as a general rule it should be weaned both for its own sake and its mother's. Usually when the child is a year old its stomach is capable of digesting solid food and may even require it. The most appropriate diet, at least for the first six or eight months after weaning, will be light wheat bread boiled in milk, used not until one or two days after baking—that made with salt or milk rising is to be preferred. The meats of young animals after boiling, eggs when properly cooked, (that is boiled for two or three minutes with the shell on), usually agree well and are highly nutritious articles of food. At the end of twelve months after the child is born, the milk of the nursing woman will usually be found to contain *colostrum*, (in this respect the secretion returning to its first character), which has a purgative effect and, if long continued, irritates the stomach and bowels of the child, injuring digestion and perverting the secretions. Thus proving positively injurious to the child, and by draining the system of the mother and overtasking the secreting powers, she becomes debilitated, her health impaired, and the foundation, so to speak, laid for more than one type of protein disease. The exceptions to the above rule, which it will be proper to make, will readily suggest themselves, or at least be easily determinated by the sensible parent, viz: If the child is very small or delicate, particularly if it is backward in teething—if the mother's milk agrees with it—if her health is good it may be best to nurse it two or three months longer. If the health of the mother is so bad that she cannot nurse her child, a healthy young woman, of cheerful temperament, should, if possible, be procured as a wet nurse, whose own child was born about the same time as the infant she takes to nurse—occasionally where the health of the mother is seemingly good and the infant does not thrive, it may be that the mother's milk does not agree with the infant—if practicable, a change of nurses may be tried in such cases, where the mother from any cause is incompetent to nurse her own child, and a suitable substitute cannot be found. The child should be fed exclusively on a little new milk fresh drawn from the cow, which should be diluted with water which has been boiled and sweetened with a little loaf sugar, until it is six or eight months old.

Children's clothes should always be loose. There should be no pins about them, for obvious reasons—it should be taught to lie on a cot or pallet without rocking—it should have a bath every morning, with water about the temperature of the surrounding atmosphere. As it grows older its exercise in the open air should be increased. Let it romp, (before it walks, allow it to crawl), don't give it "drops" or "cordials." It will rarely need other medicine than castor oil or catnip tea. The latter will usually suffice for colic, and the oil for constipation. Though neither will be seldom required if the child is properly cared for by strict attention to diet, clothing, fresh air, &c. Where the bowels are too lax, and the fecal evacuations green, a few drops of *aqua calcis*, (lime water), added to a little new milk, with gumarabic-water as a drink, (and arrow root for food, if the child is not nursing), will usually correct this condition. If the child is really sick, the parent will consult economy, by calling in an intelligent and prudent physician at the earliest practicable moment; and such a physician will usually treat the little patient with little or no drugging.

A cheerful disposition should be cultivated by the mother during lactation. The depresssing emotions greatly deteriorate the character of the lacteal secretion, anger, grief, melancholly, jealousy, etc., cause the milk to become acrid, sometimes sudden fright or violent anger, imparts to the milk a narcotic nature, so that the child may die in a few minutes after nursing, when the mother has previously been or at the time is affected by one of these emotions in a violent degree. Numerous instances might be cited of convulsions, and even death resulting from milk vitiated by violent paroxisms of grief, terror, anger, &c. They are so numerous and well attested that we can neither impute them to mere coincidence nor the credulity of those who relate them.

It is an old apothegm, "As the twig is bent, so is the tree inclined," and no less true than familiar.

An equally trite saying expressive of the same sentiment is that paradoxical expression, "the child is father to the man."

Few will contradict the assertion that the early surroundings of the child greatly influence the character of the man. However startling may be the announcement, I feel prepared to maintain the truth of the following proposition, viz: The education of the child commences for weal or woe before it can articulate—before it leaves its mother's breast—and the influences brought to bear upon the then susceptible nature of the impressible infant continue not only throughout this life, but for all eternity! The look—the gesture—the frown—the smile—the harsh expression—the kind and gentle tone of affection and love—the dark and storm clouded atmosphere—the gorgeously lighted apartments of the palace with all the luxurious surroundings of music, and other enchantments—the dark, damp and cheerless tenement of the miserable pauper surrounded with filth and wretchedness—all influence the following nature of the susceptible babe, physically, morally and mentally! How different the effect produced upon the infant ear and brain, by the course and brutal laugh of inebriates at the midnight orgie, and the sweet and gentle tones of affection. Tell me not the child is unconscious: that it comprehendeth not: that it is too young to distinguish the brutal from the refined.

Aside from the reason that example has more influence than precept, I insist that parents should ever vigilantly guard the propriety of their own conduct in the presence of their children, and ever jealously prevent any baleful influence from operating upon the forming mind of their offspring which it is in their power to control. There are certain indefinable affinities in our natures which exert upon those with whom we are in daily intercourse, particularly the young, an influence fearfully though imperceptibly potent, the manifestations of which, though unobserved, surely and silently operate with a force sufficient, in many instances, to control or direct the destiny of its recipient years after those exerting it have slumbered in the tomb. Upham and many other authorities equally high, maintain that no impression ever received through any one of the avenues (senses) to the soul is ever lost. No word, tone of voice, scenery, etc., hath ever been taken cognizance of for an instant, which is not indellibly written upon the tablets of memory, and though perhaps at the time, not understood, may by the veriest accident or most trivial circumstance in after years, possibly without the time or place, or other surroundings, be recalled when the mind bringing to its aid the acquisitions of knowledge, experience, etc., obtained subsequent to the reception of the impression—be it word or action—may interpret it, and thus renew and strengthen the impression. When these impressions are numerous and of like nature, they become as it were a part or characteristic of the individual receiving them. Hence the power of early education. In this view of our subject how fearfully responsible is the office of the parent, the guardian and instructor of the young. I would reiterate, and sound in tones of thunder that might reach the ear, awaken the attention, and touch the heart of every parent in America, that for a healthful and perfect development of the physical, mental and moral attributes of man, to the highest standard attainable, much *very much* depends upon the parents—especially the mother and her culture of the virgin child intrusted to her care so largely, should commence before it leaves her breast. It should never be deferred until it is capable of appreciating oral language. The parent commits a terrible mistake who acts or speaks in the presence of a child under the belief that the child does not comprehend. As a general thing the apprehension of a child is more acute than that of an adult, particularly with regard to the unspoken language. The faculty is with it more intuitive and hence more truthful.

